# Educational differences in cigarette smoking among adult population in Estonia, 1990–2010: does the trend fit the model of tobacco epidemic?

**DOI:** 10.1186/1471-2458-14-709

**Published:** 2014-07-10

**Authors:** Kersti Pärna, Mari-Liis Pürjer, Inge Ringmets, Mare Tekkel

**Affiliations:** 1 Department of Public Health, University of Tartu, Ravila 19, 50411 Tartu, Estonia; 2 Estonian Cancer Registry, National Institute for Health Development, Hiiu 42, 10619 Tallinn, Estonia; 3 Department of Epidemiology and Biostatistics, National Institute for Health Development, Hiiu 42, 10619 Tallinn, Estonia

**Keywords:** Daily smoking, Occasional smoking, Adults, Education, Tobacco epidemic, Estonia

## Abstract

**Background:**

In developed countries, smoking spreads through society like an epidemic in which adults from higher socioeconomic groups are the first to adopt and earlier to quit smoking, and in which exists a lag in adoption of smoking between men and women.

The objective of this study was to describe trends in daily and occasional smoking, to investigate association between smoking status and education, and to examine if the associations in 1990–2010 in Estonia fit the pattern predicted by the model of tobacco epidemic.

**Methods:**

The study was based on a 20–64-year-old subsample (n = 18740) of nationally representative postal cross-sectional surveys conducted every second year in Estonia during 1990–2010. Cigarette smoking and education were examined. χ^2^ test for trend was used to determine daily and occasional smoking trends over study years. Multinomial logistic regression model was used to test educational differences in daily and occasional smoking for every study year. Adjusted relative risk ratios (RRRs) with 95% confidence intervals were calculated.

**Results:**

In 1990–2010, daily smoking varied largely between genders showing decreasing trend among men, but not among women. In 2010, one third of men and one fifth of women were daily smokers. Daily smoking was not clearly associated with education among men in 1990–1994 and among women in 1990–2000. Men revealed inverse relationship between daily smoking and education since 1996, but women since 2002. In 2010, compared to men and women with higher education, relative risk ratio of daily smoking was 2.92 (95% CI = 2.01–4.25) among men and 2.29 (95% CI = 1.65–3.17) among women with secondary education, but 4.98 (95% CI 3.12–7.94) among men and 6.62 (95% CI = 4.07–10.76) among women with basic education.

In 1990–2010, occasional smoking was stable and similar (varying between 7–10%) among men and women, no association with education was found.

**Conclusions:**

Daily smoking patterns in Estonia fit the model of tobacco epidemic in developed countries. Educational differences in daily smoking highlight the importance of addressing smoking behaviour in the general population by educational subgroups in Estonia.

## Background

Socioeconomic inequalities in health have been studied extensively and it has been found that smoking is associated with socioeconomic position of adults [1]. In developed countries smoking prevalence is generally higher among adults from the lower socioeconomic group [2-4], while in developing countries this situation is reversed [5]. Education is often used as an indicator of socioeconomic position since it typically precedes work and income, and reflects knowledge and skills that are important for making health behaviour choices [6].

Many authors have described the lag in the adoption of smoking between higher and lower socioeconomic groups, and the lag in adoption between men and women. These aspects are described in the spread of the tobacco epidemic in societies which has followed four stages [2,5,7-10]. In the first stage, prevalence of smoking is low, and mainly a habit of higher socioeconomic groups. In the second stage, the prevalence of smoking among men increases and is similar in different socioeconomic groups. The spread of smoking among women lags 10–20 years behind that of men, and the habit is adopted first by women in the higher socioeconomic group. In the third stage, the prevalence of smoking among men decreases, as men begin to quit smoking, especially those in the higher socioeconomic group while the prevalence among women reaches a ceiling. At the end of this stage a reduction in smoking begins to be observed among women. Finally in the fourth stage, the prevalence of smoking slowly decreases both among men and women, and smoking becomes a habit concentrated mainly in the lower socioeconomic groups [2,5,7-10].

Since regaining independence from the Soviet Union in 1991, Estonia has experienced political reforms and economic changes, which, among other things, have had a strong effect on health behaviour. Smoking in Estonia has historically been similar to other post-Soviet countries where the prevalence were considerably higher for male than for female [11]. In Estonia, smoking has been analysed from different aspects [12-14]. Socioeconomic differences in smoking were analysed in 1996 and 2006 [15,16]. No in-depth analysis of long-term trends by socioeconomic position was performed in Estonia. In order to prevent and reduce tobacco consumption effectively, it is important to have knowledge of trends of smoking and socioeconomic differences in smoking in the country concerned [17-19].

The objectives of this study were to describe trends of daily and occasional smoking, to investigate association between smoking status and education, and to examine if the associations in 1990–2010 in Estonia fit the pattern predicted by the model of tobacco epidemic.

## Methods

### Data

The present study was based on the cross-sectional nationally representative postal survey of Health Behaviour among Estonian Adult Population, which is the part of the Finbalt Monitor project, conducted among 16–64-year-old adults every second year since 1990. The surveys were approved by the Tallinn Medical Research Ethics Committee. Full details of the survey methodology by the study year have been described in published reports [20-30].

### Sample and response rate

A stratified (by age, gender, and place of residence) random sample from the Estonian population aged 16–64 was ordered from the Population Register for each survey year. Initial sample size in the period 1990–2002 was 1500–2000, from the year 2004 it was 5000 persons (Table 1). The crude response rate was 72.3% in 1990 and 60.5% in 2010 being only once less than 60% (57.3% in 2006). The adjusted response rates (excluding the persons who had wrong address, left Estonia or were dead) were available only for the last four study years.

This paper studied the population aged 20–64. In 2004–2010, the crude and adjusted response rates for this age group were comparable with response rates of initial sample (Table 1). No data was available to calculate response rates for 20–64-year-olds in earlier study years.

**Table 1:** Number and response rates for the initial sample (16–64-year-olds) and response rate for the sample used in this study (20–64-year-olds) by study year in Estonia, 1990–2010

**Study year**	**Initial survey sample of 16–64-year-olds**			**Study sample of 20–64-year-olds**	
	Sample size*	**Response rate**		**Response rate**	
		**Crude**	**Adjusted**	**Crude**	**Adjusted**
1990	1500	72.3	–	–	–
1992	1500	63.2	–	–	–
1994	1500	82.9	–	–	–
1996	2000	75.4	–	–	–
1998	2000	66.1	–	–	–
2000	2000	68.8	–	–	–
2002	2000	66.9	–	–	–
2004	5000	61.5	63.4	60.1	62.8
2006	5000	57.3	59.2	56.6	58.5
2008	5000	60.1	62.2	59.7	61.9
2010	5000	60.5	62.3	60.7	62.6

*Number of persons to whom the questionnaire was sent.

### Variables

Smoking status was determined by combining answers to two questions about current and past smoking. In 1990–2002, the questionnaire included filter question “Have you ever smoked?” (yes; no) and question about current smoking “Do you currently smoke?” (yes, daily; yes, occasionally; not at all). Since 2004 two previous questions were combined “Have you ever smoked?” (no; yes, currently every day; yes, currently occasionally; yes, but I have quit). The responses to these questions served as a basis for categorising respondents as daily smokers, occasional smokers, and non-smokers (ex- and never smokers).

Education was based on the highest completed educational level and was designated as follows: basic (less than 10 school years), secondary (10–14 years), and higher education (15+ years).

### Statistical analysis

Data was analysed using statistical package Stata 11.2. Since previous studies have shown that smoking prevalence differs between men and women [3,31,32] and since this was one requirement for analysing smoking trends in relation to the model of tobacco epidemic [5], the data were analysed separately for both genders.

Prevalence of daily and occasional smoking was calculated for each study year. The directly age-standardised percentage prevalences of daily and occasional smoking were calculated using the European standard population [33]. χ^2^ test for trend was used to determine trends in daily and occasional smoking over study years.

Multinomial logistic regression model was applied to assess the association between smoking status and education for every survey year. For that, smoking status (daily smoking, occasional smoking, non-smoking) was used as a dependent variable and relative risks of daily smoking *vs* non-smoking and occasional smoking *vs* non-smoking were calculated. Education (higher, secondary, basic) was used as an explanatory variable and relative risk ratios (RRRs) with 95% confidence intervals (CI), as the measure of outcome, were calculated to compare relative risks for daily and occasional smoking in different educational groups (secondary *vs* higher education, basic *vs* higher education). The estimates were adjusted for age, ethnicity (Estonian, non-Estonian), marital status (single, married or cohabiting, divorced or separated, widowed), employment status (employed, unemployed, homemaker, student or recruited, retired).

Questionnaires which lacked information about smoking were excluded from the analysis. A total of 18740 questionnaires (7949 men and 10791 women) were used in the study (Table 2). Questionnaires with missing information concerning education (n = 151) were excluded from the analysis of smoking by education.

**Table 2:** Study sample of 20–64-year-old men and women by study year in Estonia, 1990–2010

**Study year**		**Men**		**Women**	**Total**
	**N**	**%**	**N**	**%**	**N**
1990	439	43.7	566	56.3	1005
1992	422	47.3	470	52.7	892
1994	508	43.0	673	57.0	1181
1996	611	44.2	771	55.8	1382
1998	502	44.5	627	55.5	1129
2000	467	41.3	663	58.7	1130
2002	448	41.5	631	58.5	1079
2004	1168	43.8	1500	56.2	2668
2006	1030	39.4	1584	60.6	2614
2008	1175	42.2	1607	57.8	2782
2010	1179	41.0	1699	59.0	2878
Total	7949	42.4	10791	57.6	18740

## Results

**Figure 1:**
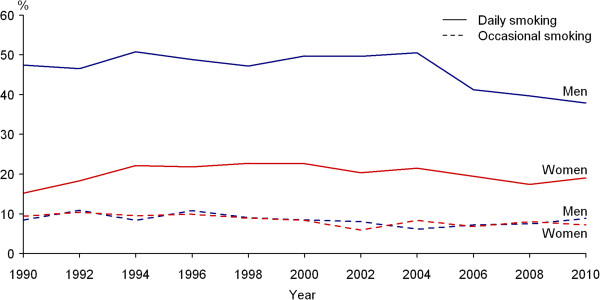
**Trends in prevalence of daily and occasional smoking among 20–64-year-olds.** Prevalence of daily smoking varied markedly between genders during the whole study period. In 1990, 47.4% of men and 15.2% of women were daily smokers. In 2010, 37.9% of men and 19.0% of women were daily smokers. The years 1990–2010 showed decreasing trend in daily smoking among men (p < 0.01). During the study period, daily smoking among women increased slightly, but this trend was not statistically significant. Occasional smoking was similar and stable among men and women during the whole study period. In 1990, 8.4% of men and 9.4% of women and in 2010, 8.8% of men and 7.2% of women were occasional smokers.

**Figure 2:**
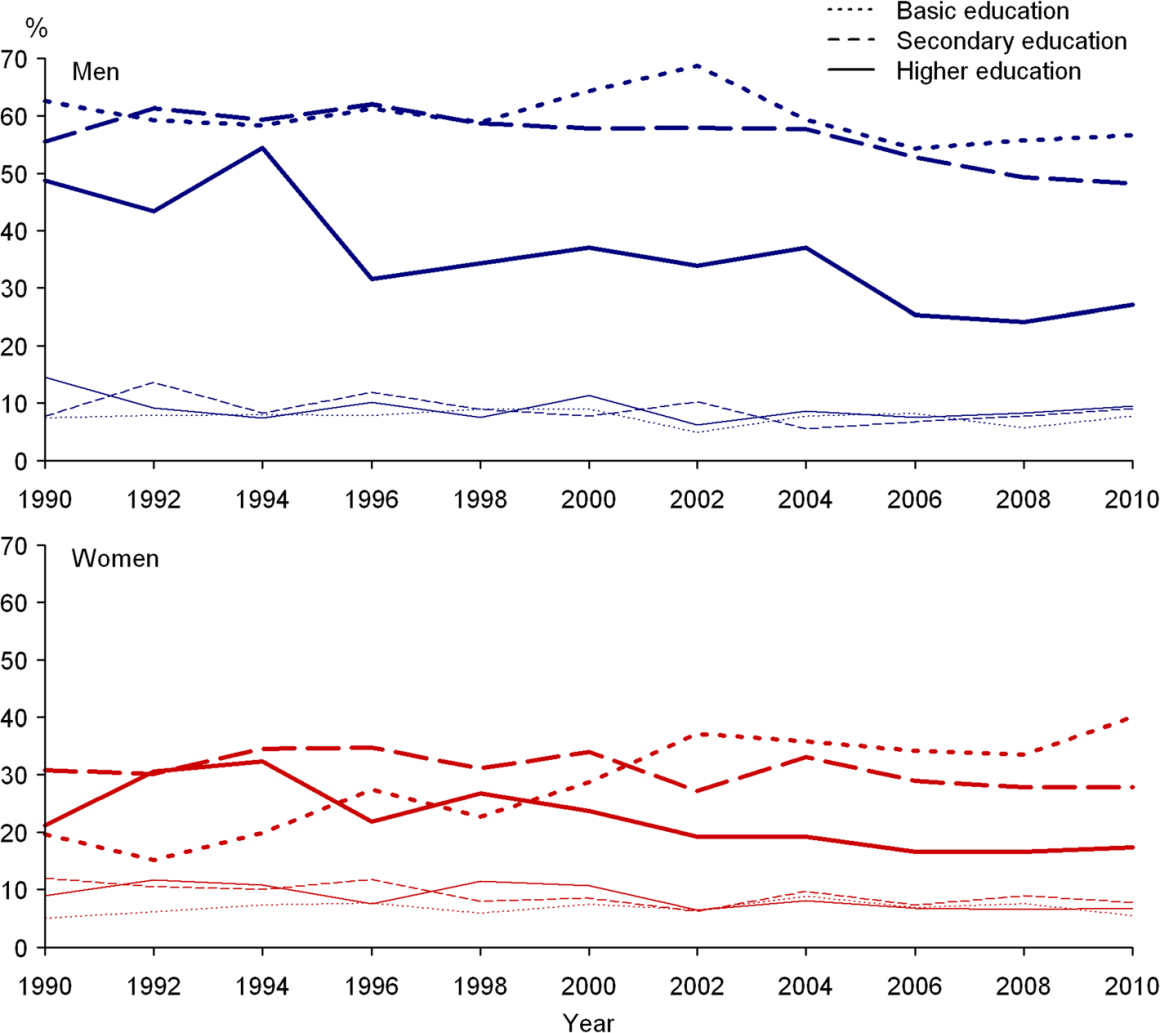
**Trends in daily (thick lines) and occasional (thin lines) smoking by education among 20–64 year-olds.** Compared to lower educated men, daily smoking was less common among men with higher education in 1990–2010, but among women since 2000. During the study period, prevalence of daily smoking decreased significantly from 48.7% to 27.1% (p < 0.01) among men with higher and from 55.5% to 48.2% (p < 0.01) among men with secondary education. Daily smoking among men with basic education decreased slightly from 62.5% to 56.6%, but the changes were not statistically significant. During the study period, the prevalence of daily smoking decreased significantly from 21.2% to 17.4% (p < 0.01) among women with higher education and doubled from 19.7% to 40.0% (p < 0.01)] among women with basic education. There were no significant changes in daily smoking among women with secondary education in 1990–2010. No significant trends over time were established in occasional smoking by education among men and women.

Prevalence of daily smoking varied markedly between genders during the whole study period (Figure 1). In 1990, 47.4% of men and 15.2% of women were daily smokers. Age-standardised prevalence (European population) of daily smoking was 47.0% and 16.7%, respectively. In 2010, 37.9% of men and 19.0% of women were daily smokers. Age-standardised prevalence of daily smoking was 38.1% and 19.5%, respectively. The years 1990–2010 showed decreasing trend in daily smoking among men (p < 0.01). During the study period, daily smoking among women increased slightly but this trend was not statistically significant.Occasional smoking was similar and stable among men and women during the whole study period (Figure 1). In 1990, 8.4% of men and 9.4% of women (age-standardised prevalence 10.3% and 11.0%, respectively), and in 2010, 8.8% of men and 7.2% of women (age-standardised prevalence 9.4% and 7.8%, respectively) were occasional smokers.During the study period, prevalence of daily smoking decreased significantly from 48.7% to 27.1% (p < 0.01) among men with higher and from 55.5% to 48.2% (p < 0.01) among men with secondary education (Figure 2). Daily smoking among men with basic education decreased slightly from 62.5% to 56.6%, but the changes were not statistically significant.In 1990–2010, the prevalence of daily smoking decreased significantly from 21.2% to 17.4% (p < 0.01) among women with higher education, but doubled from 19.7% to 40.0% (p < 0.01) among women with basic education (Figure 2). There were no significant changes in daily smoking among women with secondary education in 1990–2010. No significant trends over time were established in occasional smoking by education among men and women.

**Figure 3:**
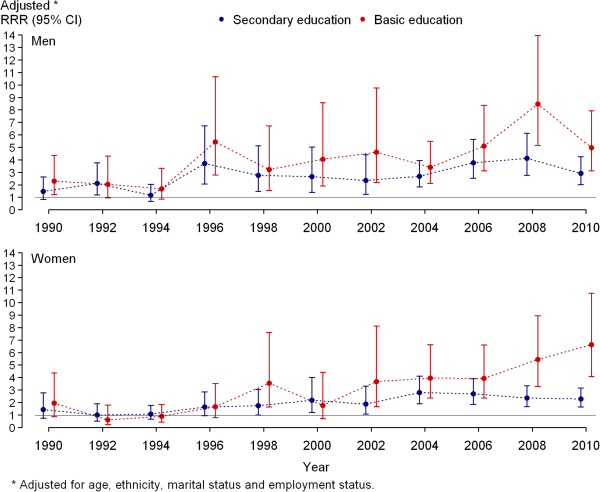
**Relative risk ratios (RRRs) of daily smoking among 20–64-year-olds by education.** No clear association was found between daily smoking and education among men in 1990–1994 and among women in 1990–2000. Men revealed inverse relationship between daily smoking and educational level since 1996, but women since 2002. Compared to men with higher education, relative risk of daily smoking (*vs* non-smoking) was 3.72 (95% CI = 2.45–6.35) times higher among men with secondary education and 5.44 (95% CI = 2.78–10.65) times higher among men with basic education in 1996. RRRs were 2.92 (95% CI = 2.01–4.25) and 4.98 (95% CI 3.12–7.94 in 2010) among men in 2010. Compared to women with higher education, relative risk of daily smoking (*vs* non-smoking) was 1.88 (95% CI = 1.07–3.31) times higher among women with secondary education and 3.67 times higher (95% CI = 1.66–8.12) among women with basic education in 2002. Respective RRRs were 2.29 (95% CI = 1.65–3.17) and 6.62 (95% CI 4.07–10.76) among women in 2010.

Using adjusted multinomial regression model, no clear association between daily smoking and education was found among men in 1990–1994 and among women in 1990–2000 (Figure 3). Men revealed inverse relationship between daily smoking and educational level since 1996, but women since 2002. Compared to men with higher education, relative risk of daily smoking (*vs* non-smoking) was 3.72 (95% CI = 2.45–6.35) times higher among men with secondary education and 5.44 (95% CI = 2.78–10.65) times higher among men with basic education in 1996. Respective RRRs were 2.92 (95% CI = 2.01–4.25) and 4.98 (95% CI 3.12–7.94 in 2010) among men in 2010. Compared to women with higher education, relative risk of daily smoking (*vs* non-smoking) was 1.88 (95% CI = 1.07–3.31) times higher among women with secondary education and 3.67 times higher (95% CI = 1.66–8.12) among women with basic education in 2002. Respective RRRs were 2.29 (95% CI = 1.65–3.17) and 6.62 (95% CI 4.07–10.76) among women in 2010.

In 1990–2010, no association between occasional smoking and education was found among men and women (data not shown).

## Discussion

This study focused on educational differences in daily and occasional smoking in 1990–2010 in Estonia. The main findings from the study were, first, that daily smoking varied largely between genders showing decreasing trend among men, but not among women. Second, the study showed that education plays an important role for daily smoking. Third, patterns of daily smoking in Estonia fit the model of tobacco epidemic in developed countries.

The survey of Health Behaviour among Estonian Adult Population present an outstanding opportunity to analyse smoking trends during two decades in Estonia because of the same study design and methodology across the whole study period.

One limitation of the survey is the use of self-reported data on smoking, which may result in underreporting of smoking. If underreporting of smoking differed by education status, the patterns found in this study may be biased. In general, self-reported smoking prevalence has been considered a valid indicator of the actual smoking status [34,35], especially in epidemiological studies. Also, studies investigating under-reporting in relation to education have shown inconsistent results [36,37]. Another limitation could be the use of education as the only indicator of socioeconomic status. On the one hand, education is skewed toward lower levels among young people, since they have not yet completed their highest level of education. On the other hand, education is being available for both men and women, including those who are currently outside employment and education usually precedes work and income. As relative risk ratios in the model were adjusted for employment status, age, ethnicity and marital status, this limitation is not likely to have affected our results. Unfortunately it was not possible to use income in this study as the question concerning the income was added to the questionnaire since 1996 only. Finally, one limitation stems from the fact that the study sample was significantly smaller until 2002 which probably reduced the power to detect significant differences. Despite these caveats, several inferences can be drawn.

The results of this study demonstrated that in 2010 one third of men and one fifth of women were daily smokers in Estonia. Notably higher smoking prevalence among men is common in the former Soviet countries [38], meanwhile in the most Scandinavian countries the smoking prevalence between genders has been diminished [31,39]. For example, 22% of men and 15% of women were daily smokers in Finland in 2011 [40]. Compared to the first study year, the prevalence of daily smoking among men decreased significantly, but slightly increased among women by 2010. The same trends in smoking among women were seen in other Baltic and former Soviet countries [11,38]. At the same time, the prevalence of smoking among women in Finland has long been steady at the level of the mid-1980s, but has shown a slight downward turn in recent years [40].

In the current study, trends in smoking by education showed that education plays an important role for daily smoking. Daily smoking started to decrease among men with higher education since 1994, these aspects showed spread of changes of daily smoking by education among women behind that of men. During the study years, the difference in daily smoking prevalence between adults with lower and higher education increased. At the same time, no educational changes in trends of occasional smoking were found. While prevalence of daily smoking among men and women with higher education decreased significantly, the same indicator among men with basic education has remained more or less on the same level but it has doubled among women during the whole study period. More educated men and women have shown decreasing trends in smoking compared with the less educated groups in many European countries (e.g. Denmark, Sweden) [3]. Main reasons why it could be harder to quit smoking among adults with lower education are that they may lack motivation, social support, enough resources to purchase nicotine replacement therapy products and also different psychological factors such as low self-esteem and susceptibility to pressure and advertising by tobacco industries [41]. Conversely, less educated men and women had greater declines in smoking than more educated adults in United Kingdom, which might be related to the pricing policy, free access to smoking cessation therapies, and restrictions on advertising of tobacco products in the country concerned [3].

Comparing the results of current study with the cigarette epidemic model, the trends in daily smoking in different educational groups in Estonia were following the same trend and smoking among women lagged that behind of men. In the beginning of the study period, daily smoking was not associated with education among men and women. Inverse relationship between smoking and education appeared among men since 1996, but among women since 2002. The similar results were found in Estonian Health Interview Surveys in 1996 and 2006. In 1996, odds to smoke daily was lower among men with higher education, but this was not the case for women [15]. In 2006, smoking was less common among men and women with higher education [16]. However, there were only two time points in Estonian Health Interview Survey, this was interview based survey and the age group was not exactly the same like in this study.

Thus, in 1990, Estonia was in the beginning of third stage of cigarette smoking model, where there were no educational differences in daily smoking among men and women. In 2010, Estonia was fitting the middle of third stage of the cigarette smoking model, where smoking was lower among both genders with higher education, but there existed lag in adoption of this between men and women. According to the educational inequalities in smoking of different countries in Europe, daily smoking in Estonia was higher among adults with lower education like in Denmark, Finland, UK, Ireland, Germany, but not like in Austria, Italy, Spain, Greece and Portugal where it was more prevalent among women with higher education [4].

## Conclusions

In 1990–2010, daily smoking varied largely between genders showing decreasing trend among men, but not among women. In 2010, one third of men and one fifth of women were daily smokers. Daily smoking was strongly associated with education among both genders. Trends of daily smoking prevalence by education during the study period and notably higher smoking among men with lower education in 1996–2010 and among women in 2002–2010 indicated that patterns of daily smoking in Estonia fit the model of tobacco epidemic in developed countries. At the same time, in 1990–2010, occasional smoking was stable and similar among men and women, and no relationship was found with education.

Educational differences in daily smoking highlight the importance of addressing smoking behaviour in the general population in Estonia by educational subgroups.

## Competing interests

The authors declare that they have no competing interests.

## Authors’ contributions

KP: made a substantial contribution to the conception and the design of the study, interpretation of the data, drafted the manuscript and has been involved in revising the manuscript critically. MLP: participated in the design of the study, has been involved in statistical analysis, drafted the manuscript and has been involved in revising the manuscript critically. IR: performed statistical analysis, has been involved in the interpretation of the data and in revising the manuscript critically. MT: principal investigator of the study of Health Behaviour among Estonian Adult Population, was involved in the interpretation of the data and critically revised the manuscript. All authors read and approved the final manuscript.

## Pre-publication history

The pre-publication history for this paper can be accessed here:

http://www.biomedcentral.com/1471-2458/14/709/prepub
